# Polyamide Noncoated Device for Adsorption-Based Microextraction and Novel 3D Printed Thin-Film Microextraction Supports

**DOI:** 10.1021/acs.analchem.1c03672

**Published:** 2022-02-03

**Authors:** Dominika Kołodziej, Łukasz Sobczak, Krzysztof Goryński

**Affiliations:** Bioanalysis Scientific Group, Faculty of Pharmacy, Collegium Medicum in Bydgoszcz at Nicolaus Copernicus University in Toruń, Jurasza 2, 85-089 Bydgoszcz, Poland

## Abstract

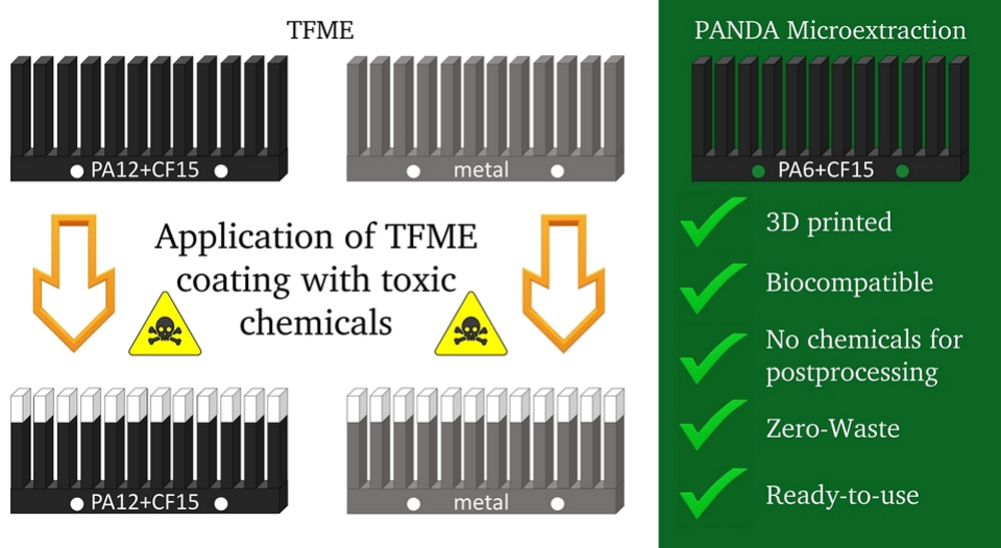

Polyamide noncoated device for adsorption-based microextraction (PANDA microextraction) is a brand new, easy to prepare, environmentally friendly, inexpensive, and efficient sample preparation method created entirely with the use of 3D printing. The proposed method is based on the extractive proprieties of the unmodified polyamide and carbon fiber blends and is compared with the highly selective thin-film microextraction (TFME). In addition, 3D printing was used to simplify the process of TFME. Prototype sample preparation devices were evaluated by the extraction of oral fluid spiked with 38 small molecules with diverse chemical natures, such as lipophilicity in the log *P* range of 0.2–7.2. The samples were analyzed by high-performance liquid chromatography coupled with tandem mass spectrometry. The results indicate that chemically and thermally resistant 3D printed supports can be successfully used as a cost-saving, environmentally friendly solution for the preparation of TFME devices, alternative to the conventional metal supports, with only marginal differences in the extraction yield (mean = 4.0%, median = 1.8%, range = 0.0–22.3%, *n* = 38). Even more remarkably, in some cases, the newly proposed PANDA microextraction method exceeded the reference TFME in terms of the extraction efficacy and offered excellent sample cleanup as favorable matrix effects were observed (mean = −8.5%, median = 7.5%, range = −34.7–20.0%, *n* = 20). This innovative approach paves the road to the simplified sample preparation with the use of emerging extractive 3D printing polymers.

3D printing emerged in the late 1980s when Charles Hull patented the Standard Tessellation Language (with the .stl file format) for the transmission and processing of 3D data files to a self-prepared prototype of a 3D printer based on stereolithography (SLA).^1^ However, it was only after Michael Cima and Emanuel Sachs incorporated fused deposition modeling (FDM), the invention of Scott Crump,^2^ into their 3D printing system that the technology was fast-tracked to mainstream use. FDM owes its success to its affordability and compatibility with an unparalleled plethora of polymers that are readily prepared as spooled filaments. The described method relies on heating the filaments to their melting point and applying the semisolid polymers layer by layer to create the designed prototype. In addition, FDM is appreciated for providing good reproducibility, as well as for the chemical and mechanical resistance of the final products. Another significant benefit is the ability to freely and instantly modify the shape and size of the prototype, all at the relatively low cost of the commercially available filaments. This multitude of benefits has resulted in the rapid expansion of FDM 3D printing into new fields, including analytical chemistry and sample preparation.^3−5^

Most often, sample preparation is a critical part of the analytical protocol and is necessary for the attainment of high-quality and unbiased results. Thus, the benefits of implementing 3D printing into analytical methodology are rapidly gaining increasing interest, well mirrored by the number of studies indexed by the phrase “3D printed” in the Web of Science database. However, papers published on the extraction devices that were prepared exclusively by the 3D printing method are still scarce. Some especially interesting examples of the fully 3D printed prototypes include the study of Su et al., who demonstrated the application of polyacrylate for the binding of trace elements in seawater to the solid-phase extraction (SPE) preconcentrator,^6^ an idea further continued by the authors with various polyurethane-based prototypes.^7^ Another research group proposed 3D printed LAYFOMM-60 (CC-Products, Germany) as a stationary phase for the extraction of small molecules.^8^ LAYFOMM-60 is a polyurethane-based thermoplastic containing water-soluble polyvinyl alcohol (PVA) that needs to be eluted with water after printing, for example, to increase the surface porosity. Published applications include the extraction of the antidiabetic drug glimepiride,^8^ extraction of endo- and exogenous steroids from plasma and phosphate-buffered saline,^9,10^ and extraction of arylpiperazine derivates of anxiolytic drugs.^11^

Another interesting idea was pursued with polybutylene terephthalate (PBT), a type of 3D printable thermoplastic material. Although not 3D printed by the authors of this study, it was proposed as a supporting material for coating with microextraction stationary phases due to its good chemical resistance and biocompatibility.^12^ PBT fibers and blades were coated with a polyacrylonitrile hydrophilic lipophilic balance (PAN-HLB) stationary phase and evaluated for the extraction of 17 doping agents from blood plasma, urine, and whole blood with good results. However, until recently, there were significant impediments that prevented the straightforward implementation of 3D prototyping in the development of microextraction-based sample preparation methods, especially with regular FDM 3D printers. The reason for these impediments was simple, yet no obvious solution was available at the time. As established through extensive method development, a preferred method for the application of microextraction coatings is spray painting, the results of which are superior to dipping or brush painting,^13^ but the preparation protocols necessitate the use of high temperature (at least 110 °C)^14^ for curing the sprayed coatings. Therefore, this requirement of good thermal resistance and good chemical resistance to the strong organic solvents that are used in the process, such as *N*,*N*-dimethylformamide (DMF), significantly hindered the 3D prototyping of the microextraction supports due to the lack of compatible and 3D printable materials. For example, thin-film microextraction (TFME) supports 3D printed from PBT would not be able to withstand the temperature of 125 °C that is used for coating with PAN-HLB^12^ or the heat wave encountered when entering the oven because of the diminished heat deflection temperature (HDT). Fortunately, this obstacle can now be overcome with recently commercialized thermoplastics such as carbon fiber-reinforced polyamides (PA + CF). These emerging biocomposites can be obtained from lignocellulosic biomass^15^ and decomposed with gentle solvent treatment utilizing nonhazardous reagents,^16^ ensuring their sustainability in addition to their already proven biocompatibility.^17,18^ Moreover, neat polyamide 6 was previously reported as a stationary phase used in SPE columns for on-line sample preparation preceding the instrumental analysis^19,20^ and for the preparation of headspace solid-phase microextraction fibers.^21,22^ The versatility and applicability of this polymer were additionally demonstrated by the authors through its application for the determination of bisphenol A contaminants in environmental waters,^23^ various insecticides in soil and waters,^24^ ochratoxin A in beer,^25^ and resveratrol in wines.^26^ Remarkably, polyamide 6 provided nearly superior results in comparison with the acclaimed octadecyl (C_18_) stationary phase.^24^ In addition, the superiority of the 3D structures prepared with polyamide 6 over the corresponding 2D structures was shown for the extraction of chlorobenzenes.^22^ However, it should be underlined that although polyamide 6 was first synthesized in 1938,^27^ all of the aforementioned studies used electrospun fibers, and until recently, the polyamides were not available as 3D printing filaments.

Moreover, the introduction of polyamides as 3D printing filaments enables pursuing more environmentally aware interests, parallel to the focus on developing and improving the analytical solutions. The principles of green analytical chemistry, emphasizing aspects such as organic solvent consumption reduction, design enabling degradation, and process sustainability, may now be impeccably implemented by combining the benefits of 3D printing and microextraction sample preparation techniques. Microextraction methods such as TFME facilitate low-volume sample analysis by combining extraction with preconcentration (occurring during the desorption step) into a single analytical protocol. Reduced sample loading with microextraction methods results in decreased organic solvent consumption in comparison with concurrent sample preparation techniques.^28,29^ Additionally, the portability and biocompatibility of microextraction methods grants unparalleled ability to perform direct on-site sampling in environmental research or in vivo sampling in medical studies, allowing simultaneous sampling and sample preparation.^30^ With ex vivo applications, biocompatibility is not only a trendy catch phrase but also has a direct impact on extraction efficacy. With biocompatible microextraction methods, extraction of the analytes from complex matrices such as blood, oral fluid, or plasma without coextraction of undesired macromolecules is possible^31^ owing to the absence of peptide and protein adsorption to the stationary phase.^32,33^ These characteristics, in conjugation with low laboratory waste production and potential for reusability of the extraction devices,^34^ demonstrate the unambiguous benefits of green analytical chemistry resulting from the replacement of the traditional sample preparation methods with microextraction while still offering comparable extraction performance.^28,29^

Furthermore, direct adsorption of the analytes to biocompatible 3D printed microextraction devices prepared with sustainable biocomposites without additional laborious pre- or postprocessing offers an unprecedented opportunity to capitalize on the benefits of microextraction techniques while simultaneously eliminating the use of any harmful reagents. For comparison, the preparation of relatively green TFME coatings still regrettably requires the use of highly toxic concentrated hydrochloric acid and DMF, a potential carcinogen and teratogen.^13^

Building upon these possibilities, we aimed to fulfill the following goals:

(1) obtain affordable and biocompatible 3D printed support for TFME devices, characterized by good chemical and thermal resistance;
(2) prepare efficient and sustainable extraction devices with 3D printed biocomposites, sparing laborious pre- or postprocessing with harmful chemicals.

Two promising blends of polyamides (nylons) with carbon fiber were selected based on their biocompatibility, high HDT, and sustainable production: polyamide 6 + carbon fiber 15% (PA6 + CF15) and polyamide 12 + carbon fiber 15% (PA12 + CF15). To the best of our knowledge, the present study introduces 3D printed TFME supports and 3D printed ready-to-use polyamide noncoated device for adsorption-based microextraction (PANDA microextraction), which do not require pre- or postprocessing with any hazardous chemical agents, for the first time.

## Experimental Section

### Preparation of Microextraction Devices

The blades used as supports for the TFME coatings were prepared from precut metal sheets (PAS Technology, Germany) and by 3D printing with a FDM method. All the supports had equal dimensions and shapes of 96-well-compatible 12-pin blades to ensure equal areas of the applied TFME coatings. Devices in the newly proposed PANDA microextraction format were prepared exclusively with 3D printing and had the same shape and size as the TFME supports.

The 3D designs were prepared in Blender version 2.82 (Free Software Foundation, Inc.) as .stl files, then sliced and converted to printer-compatible .gcode files in PrusaSlicer (Prusa Research, Czech Republic), and prototyped with a Prusa i3 MK2 printer (Prusa Research, Czech Republic) from two different types of polyamide and carbon fiber blends: 1.75 mm PA6 + CF15 (Spectrum Industrial, Spectrum Group, Poland) and 1.75 mm PA12 + CF15 (Fiberlab, Fiberlogy, Poland). The printer was fitted with a double-sided textured polyetherimide (PEI) powder-coated spring steel sheet (Prusa Research, Czech Republic) and ruby nozzle (BROZZL, Schimautz GmbH, Austria) for a 0.4 mm E3D V6 hot end. As recommended by the manufacturer, the PA6 + CF15 filament was conditioned for 2 h in an oven set at 75 °C before use. The following parameters were used for the printer: 15% linear infill on the pins of the prototypes, 15% 45° triangular infill for the remaining part of the prototypes, a heat bed temperature of 90 °C, a nozzle temperature of 260 °C, a height of 0.2 mm for the first layer, and a height of 0.05 mm for the remaining layers. A three-layer skirt outline was used. The printing speeds were 20 mm s^–1^ for the first layer, 45 mm s^–1^ for perimeters, 25 mm s^–1^ for small perimeters, 80 mm s^–1^ for solid infill, 40 mm s^–1^ for top solid infill, 30 mm s^–1^ for bridges, and 40 mm s^–1^ for the gap fill.

The metal blades were etched in concentrated hydrochloric acid (Fluka, Honeywell) for 60 min in an ultrasonic bath to increase their surface porosity. After cleaning with distilled water, the blades were dried in an oven set at 150 °C for 30 min.

A TFME coating was prepared by dispersing 10 μm C_18_-bonded silica particles with polar end-capping groups (Synergi Hydro-RP, Phenomenex) in DMF (Sigma-Aldrich, Merck Group) solution of PAN (Aldrich, Merck Group). One centimeter of the coating was applied on the tips of the blades, each consisting of 10 layers of coating slurry, utilizing a nitrogen-operated sprayer and a previously established protocol.^13^ Each layer was dried for 3 min in an oven set at 110 °C immediately after application. This temperature was previously determined to be optimal for the process.^14^

### Extraction Method

An extraction device was created by combining eight 12-pin blades to form a 96-pin brush compatible with 96-well 2 mL DeepWell plates (Nunc, Thermo Scientific). The experiments were performed with a semiautomatic plate-compatible benchtop SH10 Heater-Shaker (Ingenieurbüro CAT, Germany). Protocol included preconditioning in methanol/water (50/50, v/v; 1.5 mL, 60 min, 850 min^–1^ agitation); first rinse with water (1.5 mL, 5 s, no agitation); extraction from spiked oral fluid (1 mL, 2.5 h, 850 min^–1^ agitation); second rinse with water (1.5 mL, 5 s, no agitation); and desorption to methanol/water/formic acid (80/19.9/0.1) containing deuterium-labeled reference standards at 5 μg L^–1^ concentration (1 mL, 2 h, 850 min^–1^ agitation). Formic acid (Optima, Fisher Chemical), methanol (CHROMASOLV, Honeywell), and water (LiChrosolv, Merck Group) were all LC–MS-grade reagents. All experiments were performed in quadruplicate.

### HPLC-MS/MS Method

The extracts were analyzed by high-performance liquid chromatography coupled with tandem mass spectrometry (HPLC-MS/MS) on a Shimadzu LCMS-8060 triple quadrupole. The chromatographic method for the Agilent InfinityLab Poroshell 120 EC-C18 column (3 × 100 mm, 2.7 μm) fitted with a guard column (3 × 5 mm, 2.7 μm) was based on gradient elution with acetonitrile (CHROMASOLV, Honeywell; LC–MS grade) and water (LiChrosolv, Merck; LC–MS grade) as the mobile phases and was previously used for the separation of similar solutes.^35^ The gradient program began with 10% acetonitrile maintained for 0.5 min, succeeded by a linear increase to 100% at 26 min mark; 100% acetonitrile was maintained for 3 min, followed by rapid drop to 10% for column re-equilibration for the next 6 min. In total, the gradient program took 35 min per sample. Both mobile phases contained 0.1% formic acid, the total flow rate was 300 μL min^–1^, the injection volume was 0.7 μL, and the column temperature was maintained at 25.0 °C. The retention times and precursor–product ion transitions are listed in Table S1 in the Supporting Information.

### Oral Fluid Collection and Reference Standards

Oral fluid samples were obtained from two healthy volunteers (female aged 24 and male aged 27) in accordance with applicable regulations. These volunteers declared no previous use of the analyzed substances. The samples were pooled together to obtain a uniform matrix and spiked with a mixture of 38 reference standards, each at a 50 μg L^–1^ concentration. The spiked matrix was mixed on a benchtop shaker and stored for 60 min at room temperature to allow drug–protein binding.

Reference standards of the 38 various small molecules (log *P* calculated with the XLogP3.0 program is in the range of 0.2–7.2, and molecular masses are in the range of 149.12–528.24 Da)^36^ were purchased from LGC Standards (LGC Poland) and Sigma-Aldrich (Sigma-Aldrich Poland) as ready-to-use 1 g L^–1^ stock solutions or prepared by dissolving the powder in LC–MS-grade methanol. Deuterium-labeled reference standards of the 20 analytes were purchased from the same suppliers as 100 mg L^–1^ stock solutions or prepared from powder. A full list of reference standards is presented in Table S2 in the Supporting Information.

## Results and Discussion

This study compared the newly proposed format of PANDA microextraction with three TFME devices. Each of these TFME devices comprised three elements: a support (for the application of the coating layers), a PAN binder, and C_18_-bonded silica particles. Water-compatible polar end-capped particles were used for this study as they were previously determined to be more suitable for the aqueous samples than the conventional trimethylsilane end-capped particles.^37^ Three materials were tested as TFME supports: conventional precut metal, PA6 + CF15, and PA12 + CF15. In addition, the isolated impact of every element of the TFME devices on the extraction efficacy was investigated. Extensive results for all analytes and every factor further disclosed in this paper can be found in Table S3 in the Supporting Information.

### Data Quality

All of the 38 analyzed small molecules were successfully extracted and quantified with the HPLC-MS/MS method using both TFME and PANDA microextraction as the sample preparation techniques. The linearity and sensitivity of the HPLC-MS/MS system were verified with calibration runs of a drug-spiked desorption solvent, which resulted in at least 7-point calibration curves. The mean coefficient of determination calculated with 1/*a*^2^ weighting was *R*^2^ = 0.9998. The lowest recorded values were *R*^2^ = 0.9974 for nandrolone and *R*^2^ = 0.9981 for stanozolol. The calibration runs were performed in the 1–75 μg L^–1^ concentration range for every analyte with the exception of three steroid drugs (canrenone, nandrolone, and stanozolol), for which the quantification range was 5–75 μg L^–1^. Lower ends of the ranges were compared with previously reported limits of quantification (LOQs) for TFME methods,^38−41^ with similar or superior results obtained in this study. Full comparison is presented in Table S4 in the Supporting Information. The stability of the instrument was monitored by the system suitability test (SST) samples run in duplicate at regular intervals of 10 samples. The mean relative standard deviation (RSD) for 30 consecutive SST samples was 5.7% (median = 5.3%, min = 3.4%, max = 10.5%, *n* = 38).

### Adsorption to Noncoated TFME Supports

The method of 3D printing with FDM was shown to be perfectly suited for the preparation of TFME supports. Due to the small diameter of the extrudate that is squeezed through the nozzle, small objects such as pins of the TFME blades are composed of several parallel bundles of molten and resolidified filaments. This structure provides a porous but highly reproducible surface that does not require preprocessing with concentrated hydrochloric acid before application of the TFME coating.

Neither metal nor PA12 + CF15 adsorbs the analytes well, and this trait is desirable for the support materials. In contrast, PA6 + CF15 provides good extraction efficacy and is described in subsequent paragraphs as an alternative extraction device (PANDA microextraction) rather than a support material.

For both the metal and PA12 + CF15, the amount of the extracted analyte (from nonspecific binding) was on average just 2.8% (*n* = 38). For PA12 + CF15, the extracted amounts were in the range of 0.4–13.1% (*n* = 31), but only in the case of nine analytes were they sufficient for quantification. In the case of the metal, the extracted amounts were in the range of 0.0–21.7% (*n* = 33), but these amounts were sufficient only for the quantification of 12 of the analytes. However, two drugs (nebivolol and stanozolol) were obvious outliers contributing to the significant increase in the observed mean values. For comparison, the median values of the extracted amounts were only 0.7% (*n* = 31) for the metal and 1.2% (*n* = 33) for PA12 + CF15. Such compound-specific fluctuations, present only for a few of the analytes, exclude transfer of the small fraction of the original sample (a droplet) on the extraction device as an explanation for these results.

Adsorption to noncoated surfaces is, however, dependent on the analytes’ hydrophobicity. Below a log *P* value of 2.7 (*n* = 24),^36^ no recorded result was above the LOQ for the PA12 + CF15, and only two such results were observed for the metal [for ibutamoren (2.6%) and strychnine (2.3%)]. Above a log *P* value of 4.5 (*n* = 6),^36^ every analyte can be extracted, allowing its quantification with both noncoated supports (although with relatively poor efficacy).

### Adsorption to the PAN Binder

PAN is widely used as a biocompatible binder for immobilizing particles comprising the stationary phase of TFME devices. As such, it exhibits only weak adsorptive properties toward small molecules. Therefore, as expected, the extraction efficacies of the TFME supports coated with PAN (without C_18_-bonded particles) were marginal.

For PAN-coated metal supports, the mean extracted amount was just 3.1%. Out of 38 analytes, 3 were not detected, and 23 were below their LOQs. Therefore, only 12 analytes could be quantified with a mean extraction yield of 7.5%.

PAN-coated PA12 + CF15 supports delivered similar results. The mean extracted amount was 2.4%, with 9 analytes below the limit of detection (LOD), 20 below the LOQ, and only 9 analytes extracted in quantifiable amounts with a mean extraction yield of 5.1%.

In contrast, the PAN-coated PA6 + CF15 supports were characterized by significantly greater extraction efficacies with an average of 11.1%. No analytes were below the LOD, and only one (oxycodone) was below the LOQ. However, it should be noted that noncoated PA6 + CF15 (PANDA microextraction) exhibited significant adhesion of the small molecules, and the PAN coating only decreased the extraction efficacy by an average of 10.3% (min = 0.9%, max = 29.9%, *n* = 38). One centimeter of the coating was applied. Therefore, a small fraction of the support was immersed in the sample during extraction due to the applied agitation.

Once again, it was evident that 2 out of 38 analytes were outliers prone to nonspecific binding regardless of the contact surface. For stanozolol (log *P* = 4.5),^36^ the amount extracted with the PAN-coated metal was 18.2% (and 18.0% for noncoated metal), with a PAN-coated PA12 + CF15 extracted amount of 8.6%—falling below the LOD (and 11.1% for noncoated PA12 + CF15). For nebivolol (log *P* = 3.0),^36^ the extracted amount with the PAN-coated metal was 25.4% (21.7% for noncoated metal) and with the PAN-coated PA12 + CF15, the extracted amount was 10.3% (13.1% for noncoated PA12 + CF15).

### Impact of the TFME Support Material on the Extraction Efficacy

All compared materials (metal and both PA + CF blends) were shown to be equivalent alternatives as supports for TFME coatings, providing very similar extraction efficacies and good reproducibility of the results.

The mean TFME efficacy with the metal support was only 0.3% greater than that with the PA6 + CF15 support (median = −0.8%, min = −12.8%, max = 25.0%, *n* = 38) and only 1.0% smaller than that with the PA12 + CF15 support (median = −0.1%, min = −22.3%, max = 20.7%, *n* = 38).

**Figure 1:**
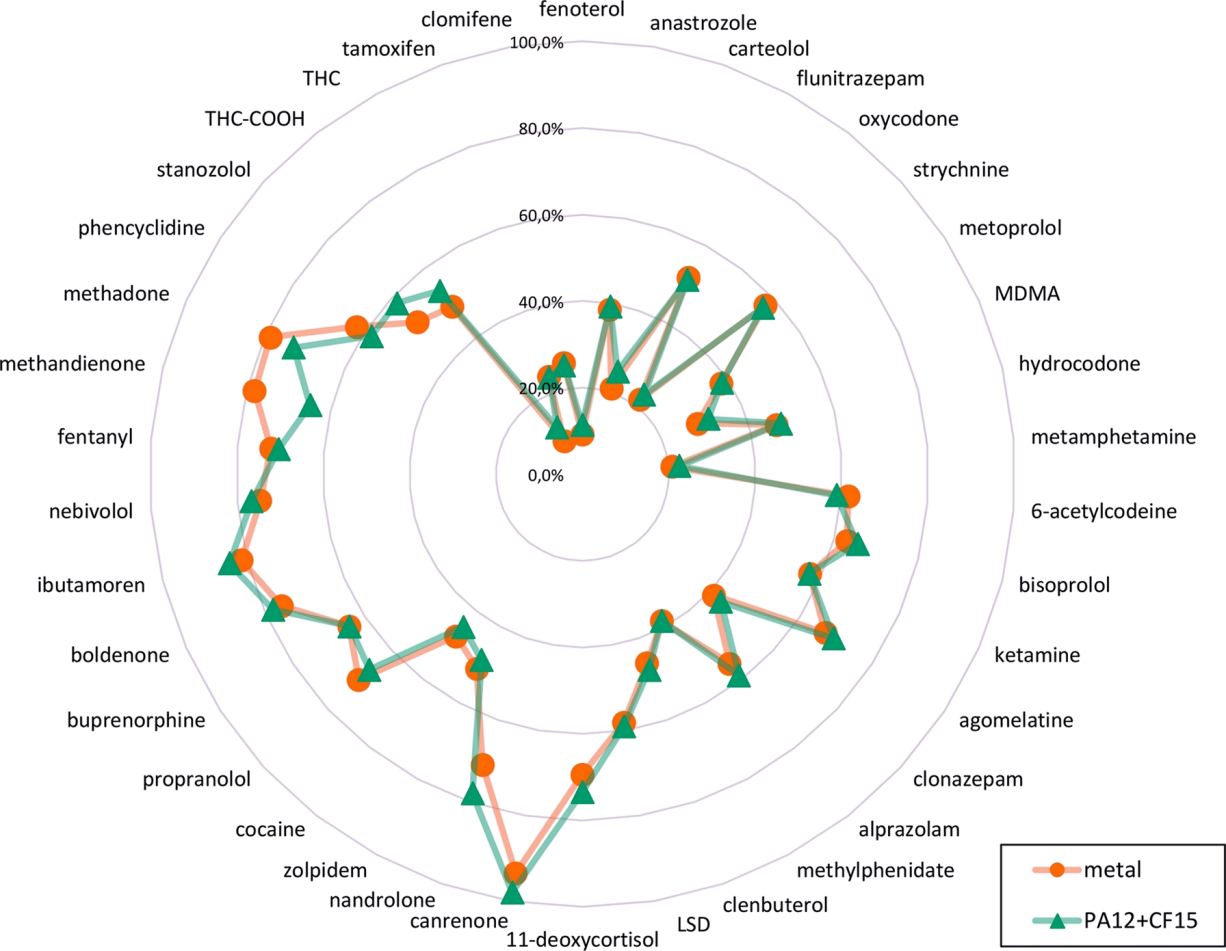
Extraction efficacies of TFME devices prepared with different support materials. Substances arranged clockwise by their log *P* value.

The mean differences in the extraction efficacies were 5.1% (median = 3.3%, min = 0.1%, max = 25.0%, *n* = 38) between the metal and PA6 + CF15 and only 4.0% (median = 1.8%, min = 0.0%, max = 22.3%, *n* = 38) between the metal and PA12 + CF15. Therefore, the PA12 + CF15 supports provided results more similar to those of the metal supports than those of the PA6 + CF15 supports. Figure 1 demonstrates the equivalence of the extraction efficacies recorded with both metal and PA12 + CF15 TFME supports. Few of the distinct exceptions, for which an above-average differences between both support materials could be observed, include more hydrophobic analytes such as synthetic opioid methadone (difference in the extraction efficacy = 5.9%, log *P* = 3.9)^36^ and three anabolic steroids: stanozolol (difference = 6.4%, log *P* = 4.5),^36^ nandrolone (difference = 7.1%, log *P* = 2.6),^36^ and methandienone (difference = 13.3%, log *P* = 3.6).^36^

The repeatability of the results recorded with all compared types of supports was very good, and only nonsignificant variations were observed. The mean RSD value for the metal support was 3.0% (median = 2.9%, min = 0.7%, max = 7.8%, *n* = 38); for PA6 + CF15, the mean RSD = 2.7% (median = 2.8%, min = 1.1%, max = 7.1%, *n* = 38); and the most favorable mean RSD value of less than 2.7% (2.68%) was recorded for PA12 + CF15 (median = 2.5%, min = 0.9%, max = 7.2%, *n* = 38).

### PANDA Microextraction—Efficacy, Linearity, and Repeatability

In this study, all analytes could be sufficiently extracted (i.e., above their levels of quantification) by PANDA microextraction with very good reproducibility (mean RSD = 2.6%, median = 2.4%, min = 0.6%, max = 5.8%, *n* = 38). These low RSD values, lower than the numbers recorded for TFME devices, result from evading the necessity of manually spray-painting the TFME coatings.

Remarkably, in addition to its versatility in allowing sufficient extraction of all the analytes in this study, PANDA microextraction exceeds TFME devices with C_18_ coatings in terms of extraction efficacies of fenoterol (log *P* = 2.0)^36^ and Δ^9^-tetrahydrocannabinol (THC; log *P* = 7.0).^36^ See Table 1 for details.

**Table 1:** Extraction Efficacies of Fenoterol and THC from Oral Fluid with Selected Microextraction Devices^a^

	extraction substance			
device	PANDA microextraction (noncoated PA6 + CF15)	C_18_-coated TFME on the PA6 + CF15 support	C_18_-coated TFME on the PA12 + CF15 support	C_18_-coated TFME on the metal support
fenoterol	28.6% (1.8%)	19.0% (4.0%)	11.3% (2.0%)	9.2% (1.3%)
THC	12.9% (4.9%)	11.2% (3.1%)	12.2% (7.2%)	8.7% (5.4%)

a Corresponding relative standard deviations given in parentheses.

The extraction efficacies for the remaining 36 substances were in the range of 4.9–60.9%, but most importantly, they were always sufficient for the quantification of every analyte from a relatively small injection volume of 0.7 μL and without any additional sample processing (such as evaporation of the solvent for preconcentration of the sample). This potentially allows the application of this method for the extraction of less stable analytes.

In comparison with the C_18_-coated TFME, PANDA microextraction provides adsorption of the analytes by the hydrophobic, hydrogen bonding, and dipole–dipole type interactions, rather than exclusively by the hydrophobic-type interactions such as octadecyl functional groups.^42^ Nevertheless, both extractive phases are best suited for the extraction of similar substances, specifically the hydrophobic multicyclic structures [with boldenone (log *P* = 3.5), canrenone (log *P* = 2.7), ibutamoren (log *P* = 1.3), methandienone (log *P* = 3.6), nandrolone (log *P* = 2.6), nebivolol (log *P* = 3.0), and propranolol (log *P* = 3.0) as mutual examples].^36^ In the case of PANDA microextraction, the best extraction efficacies were recorded for the substances in the log *P* range of 1.3–5.0 (with a mean log *P* value of 3.2, *n* = 10). With TFME, the best extraction efficacies were observed for analytes in the log *P* range of 1.3–4.0 (mean = 3.0, *n* = 10). PANDA microextraction performed worse only for the most hydrophilic ones of the target molecules, with the log *P* values in the range of 0.2–2.3 (mean = 1.8, *n* = 10). No such trend could be observed for TFME, with the worst results being for molecules with the log *P* values in the wide range of 1.0–7.2 (mean = 3.4, *n* = 10). Therefore, PANDA microextraction provided more consistent coverage of the analytes likely to the several unique adsorption mechanisms.

Adsorption and desorption to the PA6 + CF15 surface were determined as linear processes by preparing calibration curves (5–8 points, depending on extraction efficacy) from drug-spiked oral fluid samples with PANDA microextraction sample preparation in conjugation with HPLC-MS/MS analysis. The resulting coefficient of determination values, calculated with 1/*a*^2^ weighting, were in the *R*^2^ = 0.9539–0.9995 range (mean = 0.9776, median = 0.9809, *n* = 36).

### PANDA Microextraction and TFME—Sample Cleanup (Matrix Effect)

In addition, sample cleanup provided by the PANDA microextraction method was compared with that of the TFME devices prepared with three alternative support materials under comparison in this study. The degree of sample cleanup was assessed based on the differences in the signal intensities of 20 deuterium-labeled internal standards (ISs) spiked to the desorption solvent. One batch of the spiked desorption solvent was used for all extractions and preparation of the SST sample. Therefore, it was possible to demonstrate a direct relationship between the differences observed in signals measured for ISs after extraction with a given device type and for SST (mean value from four SST samples, both preceding and succeeding the extracted samples). As all extraction protocols were uniform, the degree of sample cleanup provided by a given microextraction device was the only variable accounting for the differences observed in the IS signal intensities. The degree of sample cleanup affects MS detection and is generally referred to as the matrix effect.^43^ In this study, negative matrix effect values signify signal suppression, while positive values result from signal enhancement.

For all of the compared microextraction devices, low average matrix effects were observed and only sporadically exceeded ±20% for certain drugs. All matrix effect values can be found in Table S5 in the Supporting Information. With regard to the C_18_-coated TFME devices, devices with metal support provided a mean matrix effect of −10.3% (median = −10.3%, min = −17.1%, max = −1.8%, *n* = 20); with the PA6 + CF15 support, the mean value was −15.3% (median = −14.7%, min = −47.3%, max = 0.5%, *n* = 20); and with the PA12 + CF15 support, the mean value was −10.5% (median = −11.3%, min = −19.0%, max = −3.5%, *n* = 20). For PANDA microextraction, the mean matrix effect was −8.5%, with a median value of −7.5% and a range of −34.7–20.0%, *n* = 20.

Utilizing the matrix effect to correct for the extraction efficacies of the 20 matching pairs (analyte—the IS of the analyte), one may observe that the differences between the extraction efficacies of the TFME devices prepared with metal and alternative support materials decreased even further than previously described. For the C_18_-coated PA6 + CF15, the mean difference decreased from 4.1% (median = 2.8%, min = 0.2%, max = 15.9%, *n* = 20) to 3.5% (median = 2.7%, min = 0.7%, max = 11.0%, *n* = 20). With the PA12 + CF15 support, the difference decreased from 2.7% (median = 1.4%, min = 0.1%, max = 17.4%, *n* = 20) to 2.4% (median = 1.3%, min = 0.2%, max = 13.0%, *n* = 20). Thus, additional argument for the preparation of TFME coatings with the PA12 + CF15 supports is given as its performance is similar to that prepared with the conventionally used metal supports.

### Further Discussion

**Figure 2:**
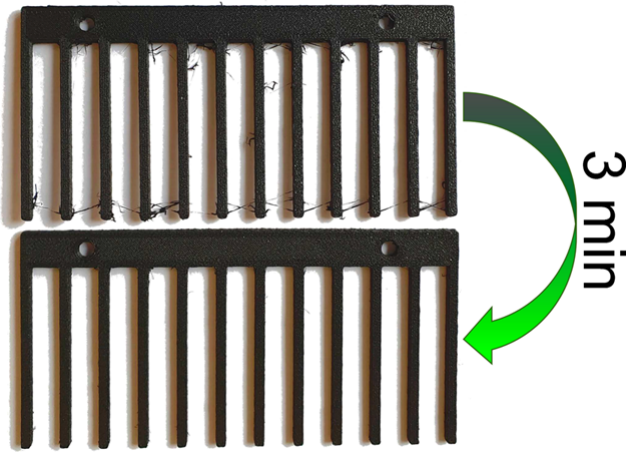
Easy and chemical-free preparation process of PANDA microextraction.

Undoubtedly, the most important part of a microextraction device is its extractive surface. PANDA microextraction, prepared entirely by 3D printing from a sustainable PA6 + CF15 blend, allows extraction by direct adsorption of the analytes to its surface. Moreover, no pre- or postprocessing with chemicals is required. Minor postprocessing only involves cutting out the remnants of the idle printer head movements (ca. 3 min, see Figure 2). Similarly, microextraction supports 3D printed from PA12 + CF15 are ready for application of the coating without prior etching in hydrochloric acid and the laborious cleaning procedure and as a result, providing cost and time savings, as well as diminished environmental impact of the preparation process.

For comparison, the preparation of conventional TFME devices required approximately 2 h for preparation of the supports, 1 h for application of the coatings, and additional time for postprocessing of the coatings after spray painting, in total over 4 h.^13^ In addition to the time consumed, the average cost of a single 12-pin TFME blade prepared for this study was 40.5 $ (33.5 $ for the coating slurry and 7 $ for the metal support) and harmful reagents such as concentrated hydrochloric acid and DMF were used in the process. With 3D printed polyamide-based TFME supports, the overall preparation time was reduced by approximately 1 h 40 min (from ca. 2 h to ca. 20 min for the preparation of the supports), and the cost was limited by 6.8 $ per single TFME blade (from 7 $ to just 0.2 $). With the presented savings, over 99% of the remaining cost is down to the cost of the coating itself. It is also worth mentioning that thanks to the identical shape, chemical and thermal resistance, as well as the adhesive and porous surface, the 3D printed supports can be chemically functionalized with any given type of microextraction coatings, just like the conventional metal supports.

In contrast to TFME, the complete process of PANDA microextraction took only 20 min (17 min for prototyping and 3 min for postprocessing), instead of over 4 h. The entire postprocessing method was chemical-free and comprised a simple single step of cutting out the remnants of the idle printer head movements. The total cost of PANDA microextraction preparation was 0.2 $ (for 0.63 m of the filament to create 2.04 g prototype of the 12-pin blade), over 200 times less than 40.5 $ for a single TFME blade. However, it must be emphasized that despite relatively high preparation/purchase costs, TFME devices are reusable, dividing the initial investment per multiple samples extracted. Additionally, less laboratory waste is generated than with alternative (e.g., protein precipitation or liquid–liquid extraction) sample preparation methods. Just like TFME, PANDA microextraction can potentially also be reused multiple times as no degradation of the device occurs during extraction or desorption with the proposed extraction protocol. If necessary, it can also be recycled without hazardous solvents.^16^ Similar to the TFME,^13,38−41^ PANDA microextraction can also be operated in semi- or fully automated high-throughput mode. Owing to the use of 96-well plates and two benchtop shakers, in this study, up to 192 samples could be processed simultaneously, resulting in less than 2 min preparation time per sample. It should also be stressed that FDM 3D printing, used to prepare both PANDA microextraction and polyamide-based TFME supports, is considered a zero-waste method due to the complete use of substrate materials (in these cases, the filament) and the lack of generated byproducts.

## Conclusions

The present study demonstrates the benefits associated with the implementation of 3D printing in analytical sample preparation. For the first time, alternatives to the costly metal supports of TFME devices are proposed. In addition, a promising new PANDA microextraction format is introduced. These advances were materialized utilizing novel carbon fiber-reinforced polyamide biocomposites, which are both sustainable^15,16^ and biocompatible.^17,18^

Both TFME and PANDA microextraction methods are compatible with 96-well plates, allowing the simultaneous processing of multiple samples. The new TFME supports prepared with PA12 + CF15 are equivalent to the conventionally used metal supports. However, their introduction helps preserve the environment, financial resources, and time. In turn, PANDA microextraction provides not only a reduction in production costs (ca. 200 times) and time savings (over 12 times) but also excellent sample cleanup, good extraction efficacy, and reproducibility—in the case of some analytes, these qualities were superior even to those of the established and highly selective TFME method. PANDA microextraction, prepared with a PA6 + CF15 biocomposite, is ready to use after prototyping and only a brief postprocessing step, which is a significant improvement over the polyurethane-based LAYFOMM-60 first proposed for 3D printed extraction devices. According to the recommendations of the manufacturer, LAYFOMM-60 requires a 2–4 day preconditioning protocol to elute water-soluble PVA before it is ready to use, especially to minimize matrix effects when samples are analyzed by HPLC-MS. PANDA microextraction only requires cutting out the remnants of the idle printer head movements. Therefore, the entire preparation process is free of any reagents (particularly, concentrated hydrochloric acid and DMF). In addition to the previously mentioned benefits and savings, PANDA microextraction can be shared as a ready-to-print file and prepared with a portable 3D printer on-site immediately before its use. No specialized laboratory equipment or good technical skills are necessary for the process. Unlike spray painting of handmade TFME coatings, with its outcome highly dependent on the practical and manual experience of the personnel preparing the device (10 layers of dense coating slurry are applied on both sides of the supports with a hand sprayer), additional validation steps are needed to ensure adequate repeatability of the product.

As demonstrated in this study, PANDA microextraction offers unique advantages that can be applied in sample preparation for numerous structurally diverse small molecules: doping agents, endogenous hormones, prohibited substances, and therapeutic drugs. In addition, biocompatibility enables direct application in in vivo studies (e.g., saliva sampling) and simplifies the analytical protocol, possibly allowing analysis of the most labile analytes. Ultimately, this study introducing brand new sample preparation method paves the road to the future application of emerging extractive 3D printing polymers to encourage a new direction in general analytical chemistry.

## Supporting Information Available

Monitored precursor–product ion(s) transitions; list of reference standards in the alphabetical order; extraction efficacies [%] of evaluated microextraction devices and their elements; comparison of the presented method with previously published limits of quantification [in μg L^–1^] for TFME methods; and matrix effects [%] determined for the evaluated microextraction devices and their elements (PDF)
