# Supplementary material for: Polyamide Noncoated Device for Adsorption-Based Microextraction and Novel 3D Printed Thin-Film Microextraction Supports
Source: Anal Chem. 2022 Feb 3;94(6):2764–71. doi: 10.1021/acs.analchem.1c03672 (PMC8851416; doi:10.1021/acs.analchem.1c03672)
Supplement: Supplementary file 1 — ac1c03672_si_001.pdf [file ac1c03672_si_001.pdf]

Supporting Information for

Polyamide Noncoated Device for Adsorption-Based Microextraction and Novel 3D Printed Thin-Film Microextraction Supports

by Dominika Kołodziej, Łukasz Sobczak, and Krzysztof Goryński*

Bioanalysis Scientific Group, Faculty of Pharmacy, Collegium Medicum in Bydgoszcz at Nicolaus Copernicus University in Toruń, Jurasza 2, 85-089 Bydgoszcz, Poland; Email: gorynski@cm.umk.pl

Table of Contents:

Table S1. Monitored precursor – product ion(s) transitions.
Table S2. List of reference standards in alphabetical order.
Table S3. Extraction efficacies [%] of evaluated microextraction devices, and their elements.
Table S4. Comparison of presented method with previously published limits of quantification [in μg L^{-1}] for Thin-Film Microextraction methods.
Table S5. Matrix effects [%] determined for evaluated microextraction devices, and their elements.

Table S1. Monitored precursor – product ion(s) transitions.

substance	retention time [min]	precursor ion [m/z]	product ions:					
			1 [m/z]	CE [V]	2 [m/z]	CE [V]	3 [m/z]	CE [V]
fenoterol	4.156	304.00	107.15	-31	135.15	-18	286.10	-14
carteolol	4.499	293.00	237.15	-16	202.15	-21	74.10	-23
oxycodone D_3	4.532	319.00	301.20	-20	244.10	-30	259.20	-27
oxycodone	4.567	316.10	298.20	-19	241.20	-29	256.10	-26
hydrocodone D_3	4.964	303.00	199.10	-31	171.15	-40	128.00	-55
hydrocodone	4.914	300.00	199.15	-30	171.10	-40	128.15	-55
methamphetamine D_5	5.023	154.90	92.10	-20	91.10	-20	121.20	-14
methamphetamine	5.036	150.00	91.10	-21	65.10	-41	119.20	-15
MDMA D_5	5.227	198.90	165.10	-14	107.15	-24	135.20	-20
MDMA	5.242	194.00	163.10	-13	105.10	-24	133.10	-20
strychnine	5.430	334.90	184.10	-37	156.20	-46	129.15	-55
ketamine D_4	5.732	242.00	129.05	-28	224.10	-15	211.10	-15
ketamine	5.711	237.90	125.05	-26	220.10	-14	207.15	-14
metoprolol	6.499	267.90	116.15	-19	74.15	-22	72.10	-20
6-acetylcodeine	6.911	342.00	225.15	-27	165.15	-47	197.20	-31
clenbuterol D_9	6.648	286.00	204.10	-18	268.20	-12	169.05	-30
clenbuterol	6.681	277.10	203.05	-16	132.10	-28	168.15	-30
methylphenidate	6.765	234.00	84.15	-20	56.10	-46	91.10	-46
zolpidem D_6	7.517	314.00	235.10	-36	236.20	-29	263.20	-27
zolpidem	7.537	307.90	235.15	-36	236.20	-28	263.20	-26
cocaine	7.501	303.90	182.20	-20	82.10	-30	105.10	-33
cocaine D_3	7.503	307.00	185.20	-20	77.10	-55	85.20	-30
LSD D_3	7.878	327.00	226.20	-25	210.20	-44	208.15	-29
LSD	7.844	323.90	223.20	-24	208.15	-28	207.10	-42
bisoprolol	8.018	326.20	116.20	-19	74.05	-26	72.10	-25
phencyclidine D_5	8.801	249.00	96.15	-32	86.15	-15	164.25	-14
phencyclidine	8.828	244.00	91.05	-30	86.10	-15	159.20	-14
propranolol D_7	8.912	267.00	189.20	-18	117.20	-19	161.20	-26
propranolol	8.980	260.10	116.20	-19	183.10	-18	155.20	-26
fentanyl D_5	9.325	342.10	188.20	-24	105.10	-39	103.05	-55
fentanyl	9.364	337.00	188.20	-23	105.15	-37	103.10	-55
buprenorphine	9.891	468.10	55.15	-54	396.25	-39	414.25	-35
buprenorphine D_4	9.895	472.10	59.15	-52	400.20	-42	415.25	-37
ibutamoren	10.604	529.00	267.10	-21	91.15	-52	263.15	-18
nebivolol	11.348	406.10	151.10	-31	123.10	-42	103.10	-55
alprazolam D_5	11.420	314.10	210.20	-42	286.10	-27	279.25	-27
alprazolam	11.484	308.90	205.15	-28	281.15	-40	274.10	-26
anastrozole	11.506	294.00	225.20	-22	210.20	-34	115.05	-55
stanozolol	11.745	329.10	81.15	-46	95.15	-41	121.10	-38
methadone D_3	11.585	313.10	268.15	-16	105.05	-28	77.15	-55
methadone	11.622	310.00	265.15	-15	105.10	-27	77.05	-55
11-deoxycortisol	11.617	347.15	97.10	-30	109.05	-32	79.05	-50
boldenone	12.042	287.00	121.20	-22	135.20	-15	77.15	-55
clonazepam	12.062	316.00	270.10	-25	214.05	-38	207.15	-34
clonazepam D_4	12.108	319.90	274.15	-26	218.15	-37	211.20	-34
agomelatine	12.210	244.00	185.20	-16	170.10	-27	141.10	-47
nandrolone	12.528	275.10	109.10	-27	239.10	-18	257.30	-17
methandienone	12.691	301.20	121.05	-24	149.25	-15	77.00	-55
flunitrazepam	12.851	313.90	268.15	-27	239.10	-34	183.10	-53
flunitrazepam D_7	12.861	321.10	275.15	-27	246.15	-37	245.10	-37
clomifene	15.133	406.10	100.15	-25	72.10	-35	58.15	-39
tamoxifen	15.607	372.00	72.10	-24	70.10	-46	129.15	-27
canrenone	15.153	341.00	107.15	-30	187.25	-21	235.15	-25
canrenone D_6	15.204	347.20	107.20	-31	189.20	-24	91.10	-54
THC-COOH D_3	19.956	348.00	330.20	-17	302.25	-20	196.25	-27
THC-COOH	19.982	345.00	327.10	-16	299.25	-21	193.30	-26
THC D_3	24.938	317.80	195.05	-19	235.95	-10	85.05	-35
THC	24.968	315.00	193.10	-22	123.10	-32	259.15	-19

Substances arranged by retention order. CE = collision energy.

Table S2. List of reference standards in alphabetical order.

reference standard	stock solution	grade	manufacturer
(+\-)-3,4-methylenedioxy methamphetamine (MDMA)	MeOH solution 1 g L^{-1}	CRM (primary standard)	LGC
6-acetylcodeine	ACN solution 1 g L^{-1}	CRM (primary standard)	Cerillant
11-deoxycortisol	MeOH solution 1 g L^{-1}	CRM (primary standard)	Cerillant
(-)-11-nor-9-carboxy- Δ^9- tetrahydrocannabinol (THC-COOH)	MeOH solution 1 g L^{-1}	CRM (primary standard)	LGC
agomelatine	MeOH solution from powder 1 g L^{-1}	N/A	TRC
alprazolam	MeOH solution 1 g L^{-1}	CRM (primary standard)	LGC
anastrozole	MeOH solution from powder 1 g L^{-1}	reference standard (100%)	LGC
bisoprolol	MeOH solution 1 g L^{-1}	CRM (primary standard)	LGC
boldenone	MeOH solution from powder 1 g L^{-1}	analytical standard (≥ 98%)	VETRANAL™ (Sigma-Aldrich)
buprenorphine	MeOH solution 1 g L^{-1}	CRM (primary standard)	Cerillant
canrenone	MeOH solution from powder 1 g L^{-1}	HPLC (≥ 97%)	Sigma
carteolol	MeOH solution from powder 1 g L^{-1}	USP reference standard (100%)	USP
clenbuterol	DMSO solution 1 g L^{-1}	CRM (primary standard)	LGC
clomifene	MeOH solution from powder 1 g L^{-1}	analytical standard	Sigma-Aldrich
clonazepam	MeOH solution 1 g L^{-1}	CRM (primary standard)	Cerillant
cocaine	ACN solution 1 g L^{-1}	CRM (primary standard)	LGC
fenoterol	MeOH solution from powder 1 g L^{-1}	reference standard (99.9%)	LGC
fentanyl	MeOH solution 1 g L^{-1}	CRM (primary standard)	LGC
flunitrazepam	MeOH solution 1 g L^{-1}	CRM (primary standard)	LGC
hydrocodone	MeOH solution 1 g L^{-1}	CRM (primary standard)	Cerillant
ibutamoren	MeOH solution from powder 1 g L^{-1}	HPLC (≥ 98%)	LGC
ketamine	MeOH solution 1 g L^{-1}	CRM (primary standard)	LGC
lysergic acid diethylamide (LSD)	ACN solution 1 g L^{-1}	CRM (primary standard)	LGC
methadone	MeOH solution 1 g L^{-1}	CRM (primary standard)	Cerillant
methandienone	1,2-dimethoxyethane solution 1 g L^{-1}	CRM (primary standard)	Cerillant
methamphetamine	MeOH solution 1 g L^{-1}	CRM (primary standard)	LGC
methylphenidate	MeOH solution 1 g L^{-1}	CRM (primary standard)	LGC
metoprolol	MeOH solution 1 g L^{-1}	CRM (primary standard)	LGC
nandrolone	ACN solution 1 g L^{-1}	CRM (primary standard)	LGC
nebivolol	MeOH solution from powder 1 g L^{-1}	HPLC (≥ 98%)	Sigma-Aldrich
oxycodone	MeOH solution 1 g L^{-1}	CRM (primary standard)	LGC
phencyclidine	MeOH solution 1 g L^{-1}	CRM (primary standard)	LGC
propranolol	MeOH solution 1 g L^{-1}	CRM (primary standard)	LGC
stanozolol	ACN solution 1 g L^{-1}	CRM (primary standard)	LGC
strychnine	MeOH solution from powder 1 g L^{-1}	HPLC (≥ 98%)	Sigma-Aldrich
tamoxifen	MeOH solution from powder 1 g L^{-1}	analytical standard (> 98%)	Sigma-Aldrich
(-)-Δ^9-tetrahydrocannabinol (THC)	MeOH solution 1 g L^{-1}	CRM (primary standard)	LGC
Zolpidem	MeOH solution 1 g L^{-1}	CRM (primary standard)	LGC
deuterium-labelled internal standards			
(+\-)-3,4-methylenedioxy methamphetamine D_5 (MDMA D_5)	MeOH solution 100 mg L^{-1}	CRM (primary standard)	Cerillant
(-)-11-nor-9-carboxy- Δ^9- tetrahydrocannabinol D_3 (THC-COOH D_3)	MeOH solution 100 mg L^{-1}	CRM (primary standard)	Cerillant
alprazolam D_5	MeOH solution 100 mg L^{-1}	CRM (primary standard)	Cerillant
buprenorphine D_4	MeOH solution 100 mg L^{-1}	CRM (primary standard)	Cerillant
canrenone D_6	MeOH solution from powder 100 mg L^{-1}	99,85%	LGC
clenbuterol D_9	MeOH solution 100 mg L^{-1}	CRM (primary standard)	Cerillant
clonazepam D_4	MeOH solution 100 mg L^{-1}	CRM (primary standard)	Cerillant
cocaine D_3	ACN solution 100 mg L^{-1}	CRM (primary standard)	Cerillant
fentanyl D_5	MeOH solution 100 mg L^{-1}	CRM (primary standard)	Cerillant
flunitrazepam D_7	MeOH solution 100 mg L^{-1}	CRM (primary standard)	LGC
hydrocodone D_3	MeOH solution 100 mg L^{-1}	CRM (primary standard)	Cerillant
ketamine D_4	MeOH solution 100 mg L^{-1}	CRM (primary standard)	LGC
lysergic acid diethylamide D_3 (LSD D_3)	ACN solution 100 mg L^{-1}	CRM (primary standard)	Cerillant
methamphetamine D_5	MeOH solution 100 mg L^{-1}	CRM (primary standard)	Cerillant
methadone D_3	MeOH solution 100 mg L^{-1}	CRM (primary standard)	Cerillant
oxycodone D_3	MeOH solution 100 mg L^{-1}	CRM (primary standard)	Cerillant
phencyclidine D_5	MeOH solution 100 mg L^{-1}	CRM (primary standard)	Cerillant
propranolol D_7	MeOH + 5% 1M HCl solution 100 mg L^{-1}	CRM (primary standard)	Cerillant
(-)-Δ^9-tetrahydrocannabinol D_3 (THC D_3)	MeOH solution 100 mg L^{-1}	CRM (primary standard)	Cerillant
zolpidem D_6	MeOH solution 100 mg L^{-1}	CRM (primary standard)	Cerillant

Substances arranged by retention order. MeOH = methanol; ACN = acetonitrile; CRM = certified reference material.

Table S3. Extraction efficacies [%] of evaluated microextraction devices, and their elements.

substance \ support	TFME blades with C_{18} coating			TFME blades with no coating			TFME blades with PAN-only coating		
	PA6+CF15	PA12+CF15	metal	PA6+CF15 (PANDA Microextraction)	PA12+CF15	metal	PA6+CF15	PA12+CF15	metal
fenoterol	19.0 (4.0)	11.3 (2.0)	9.2 (1.3)	28.6 (1.8)	0.7 (9.7) *	0.4 (42.6) *	18.8 (5.8)	0.6 (10.4) *	0.3 (8.3) *
carteolol	23.3 (3.9)	25.1 (3.4)	20.9 (2.2)	12.2 (1.7)	0.7 (9.1) *	0.5 (48.6) *	5.2 (7.3)	0.6 (14.7) *	0.4 (8.7) *
oxycodone	5.9 (3.1)	23.2 (3.8)	21.7 (7.8)	2.1 (2.8)	0.6 (12.9) *	0.4 (35.5) *	1.2 (9.3) *	0.6 (14.3) *	0.4 (11.2) *
hydrocodone	35.9 (3.7)	47.4 (2.3)	46.4 (4.1)	6.3 (3.7)	0.8 (13.1) *	0.6 (37.3) *	3.0 (7.4)	0.8 (15.7) *	0.6 (11.2) *
methamphetamine	21.1 (2.9)	22.5 (2.9)	20.9 (5.5)	5.7 (1.1)	0.6 (8.9) *	0.5 (41.1) *	3.1 (8.1)	0.6 (12.2) *	0.4 (8.1) *
MDMA	30.0 (2.9)	31.8 (3.7)	29.1 (4.5)	10.2 (1.3)	0.8 (8.4) *	0.7 (35.7) *	5.2 (7.0)	0.8 (13.2) *	0.6 (8.6) *
strychnine	46.4 (3.1)	56.6 (3.5)	57.6 (3.3)	16.2 (1.7)	< LOD	2.3 (14.8)	7.6 (8.8)	< LOD	2.4 (17.3)
ketamine	56.7 (2.2)	57.3 (3.7)	57.5 (4.4)	8.8 (2.2)	0.9 (2.5) *	0.6 (33.6) *	4.1 (6.8)	0.9 (17.3) *	0.6 (8.8) *
metoprolol	28.6 (3.3)	38.5 (4.1)	38.4 (1.7)	5.1 (2.1)	0.6 (13.2) *	0.5 (53.0) *	2.7 (8.2)	0.6 (22.1) *	0.4 (22.0) *
6-acetylcodeine	36.7 (1.4)	59.0 (1.9)	61.7 (2.9)	5.8 (2.5)	0.7 (7.9) *	0.7 (31.3) *	2.8 (4.0)	0.7 (18.3) *	0.8 (12.8) *
clenbuterol	45.3 (3.3)	47.8 (2.4)	46.2 (4.7)	21.2 (0.8)	1.0 (4.9) *	0.9 (22.5) *	10.6 (8.4)	1.0 (12.7) *	0.9 (13.6) *
methylphenidate	38.5 (2.3)	38.5 (2.7)	38.6 (3.8)	4.9 (2.6)	0.4 (9.8) *	0.4 (29.9) *	2.4 (9.6)	0.5 (18.9) *	0.5 (12.5) *
zolpidem	48.2 (2.1)	48.9 (1.7)	51.1 (3.0)	16.2 (1.2)	< LOD	1.2 (9.2) *	6.2 (6.0)	< LOD	1.3 (22.8) *
cocaine	43.5 (3.6)	44.8 (3.5)	47.5 (4.6)	5.2 (3.4)	0.5 (6.7) *	0.4 (32.4) *	2.8 (9.5)	0.6 (14.3) *	0.5 (8.5) *
LSD	57.9 (2.3)	59.1 (2.1)	58.3 (2.4)	23.6 (2.3)	0.9 (12.8) *	1.0 (17.9) *	9.0 (5.5)	0.9 (13.0) *	1.1 (23.4) *
bisoprolol	61.9 (3.2)	65.6 (1.2)	63.1 (3.1)	6.0 (1.4)	0.8 (2.7) *	0.6 (41.7) *	3.0 (10.9)	0.7 (11.6) *	0.5 (7.2) *
phencyclidine	58.5 (2.8)	58.2 (2.4)	62.2 (2.9)	17.4 (2.8)	0.9 (8.6) *	1.0 (14.5) *	9.2 (5.9)	1.1 (18.0) *	1.7 (11.8) *
propranolol	62.4 (3.7)	66.8 (1.5)	70.2 (3.1)	32.9 (2.2)	2.8 (4.9)	2.8 (5.1)	22.0 (8.0)	2.5 (15.8)	3.7 (13.3)
fentanyl	70.2 (2.7)	70.4 (1.6)	72.1 (2.2)	27.1 (2.3)	2.0 (10.2) *	2.9 (3.1)	14.3 (5.3)	2.2 (20.6)	3.8 (21.0)
buprenorphine	62.1 (2.5)	64.3 (1.6)	64.3 (0.7)	34.6 (2.9)	6.3 (4.8)	3.3 (10.5)	18.9 (7.9)	6.8 (23.1)	5.2 (6.0)
ibutamoren	84.0 (2.2)	84.1 (2.1)	81.2 (2.9)	36.4 (2.6)	1.2 (5.9) *	2.6 (14.9)	15.4 (8.0)	< LOD	4.1 (10.2)
nebivolol	77.3 (1.8)	76.7 (1.0)	74.7 (0.7)	60.9 (4.3)	13.1 (5.6)	21.7 (9.8)	43.3 (5.2)	10.3 (18.6)	25.4 (5.2)
alprazolam	58.3 (2.0)	58.9 (2.8)	55.4 (3.5)	10.9 (5.8)	< LOD	< LOD	4.1 (2.3)	< LOD	1.3 (13.3) *
anastrozole	39.3 (3.1)	39.2 (1.8)	38.5 (3.7)	7.1 (0.6)	< LOD	< LOD	2.7 (12.6)	< LOD	0.8 (15.3) *
stanozolol	59.0 (2.6)	58.2 (4.7)	51.8 (3.7)	45.7 (3.6)	11.1 (15.6)	18.0 (10.5)	35.6 (19.3)	8.6 (13.2) *	18.2 (8.9)
methadone	73.1 (3.0)	72.8 (1.5)	78.7 (2.3)	26.9 (1.5)	1.9 (14.4) *	3.8 (4.2)	15.7 (8.0)	3.1 (31.0)	5.9 (14.3)
11-deoxycortisol	74.9 (3.0)	73.5 (2.7)	69.4 (1.9)	28.6 (2.2)	< LOD	0.0*	10.6 (9.8)	< LOD	< LOD
boldenone	80.7 (2.3)	78.0 (1.1)	75.9 (2.1)	32.2 (2.8)	1.8 (7.2) *	< LOD	12.9 (9.1)	1.6 (5.3) *	< LOD
clonazepam	46.1 (2.8)	43.6 (2.7)	41.3 (2.2)	24.4 (3.0)	1.3 (16.6)	0.5 (27.2) *	9.2 (3.9)	1.1 (12.1) *	0.4 (10.3) *
agomelatine	72.8 (2.3)	69.4 (2.6)	67.1 (2.0)	42.4 (1.6)	4.4 (5.0)	0.6 (40.8) *	19.3 (6.1)	3.5 (14.4)	0.6 (5.5) *
nandrolone	83.8 (7.1)	78.0 (3.2)	70.9 (2.9)	33.5 (1.8)	< LOD	0.0*	15.1 (15.7)	0.0*	0.0*
methandienone	85.6 (1.6)	64.8 (1.5)	78.1 (0.8)	31.5 (1.8)	1.4 (9.2) *	< LOD	12.7 (8.3)	< LOD	0.8 (8.3) *
flunitrazepam	54.0 (1.1)	51.0 (4.7)	51.5 (3.5)	11.8 (2.5)	< LOD	< LOD	4.6 (6.4)	< LOD	< LOD
clomifene	28.4 (1.6)	25.5 (3.5)	26.0 (3.0)	24.7 (5.3)	6.9 (11.1)	8.6 (6.7)	16.0 (7.3)	5.5 (13.9)	7.8 (6.7)
tamoxifen	25.0 (1.9)	23.5 (3.5)	23.8 (2.6)	20.0 (3.9)	5.3 (11.5)	8.3 (7.3)	12.5 (7.0)	4.4 (12.7)	7.8 (6.3)
canrenone	100.0 (1.8)	98.1 (2.5)	93.5 (1.9)	56.7 (3.3)	2.9 (10.9) *	0.0	26.9 (4.4)	2.7 (10.6) *	< LOD
THC-COOH	53.8 (1.7)	53.6 (0.9)	49.0 (2.1)	15.3 (3.0)	2.3 (8.7)	2.5 (13.2)	5.8 (3.5)	1.9 (12.7) *	3.5 (9.1)
THC	11.2 (3.1)	12.2 (7.2)	8.7 (5.4)	12.9 (4.9)	11.1 (6.5)	2.4 (23.1)	7.8 (7.6)	8.0 (8.3)	2.4 (6.3)

Corresponding relative standard deviation values [%] given in parentheses, substances arranged by retention order, n = 4, *indicates results below the established LOQ (5 µg L^{-1} for canrenone, nandrolone, and stanozolol; 1 µg L^{-1} for all other substances).

Table S4. Comparison of the presented method with previously published limits of quantification [in µg L^{-1}] for Thin-Film Microextraction methods.

reference	this study	Boyacı, et al., *Anal. Chim. Acta* **2014**, *809*, 69-81.	Vasiljevic et al., *Rapid Commun. Mass Spectrom.* **2019**, *33*, 1423-1433.		Goryński et al., *J. Pharm. Biomed. Anal.* **2016**, *127*, 147-155.		Reyes-Garcés et al., *J. Chromatogr. A* **2014**, 1374, 40-49.
matrix / substance	oral fluid	urine	urine	plasma	urine	plasma	plasma
6-acetylocodeine	1	5	-	-	-	-	-
anastrozole	1	5	-	-	-	-	-
bisoprolol	1	5	-	-	-	-	0.5
buprenorphine	1	5	-	-	-	-	-
canrenone	5	5	-	-	-	-	-
clenbuterol	1	15	-	-	-	-	0.5
cocaine	1	5	-	-	-	-	-
fenoterol	1	1	-	-	0.23	0.39	-
fentanyl	1	5	0.5	1	-	-	-
hydrocodone	1	-	25	2.5	-	-	-
methadone	1	-	2.5	10	-	-	-
methamphetamine	1	5	-	-	-	-	0.5
metoprolol	1	5	-	-	-	-	0.25
nandrolone	5	10	-	-	-	-	-
oxycodone	1	5	25	25	-	-	-
propranolol	1	5	-	-	-	-	1
stanozolol	5	10	-	-	-	-	0.5
strychnine	1	10	-	-	-	-	1
THC	1	5	-	-	-	-	-

Substances arranged in alphabetical order.

Table S5. Matrix effects [%] determined for evaluated microextraction devices, and their elements.

substance \ support	TFME blades with C_{18} coating			TFME blades with no coating			TFME blades with PAN-only coating		
	PA6+CF15	PA12+CF15	metal	PA6+CF15 (PANDA Microextraction)	PA12+CF15	metal	PA6+CF15	PA12+CF15	metal
oxycodone	-47.3 (1.9)	-3.6 (2.2)	-1.8 (0.9)	-33.5 (2.3)	10.0 (4.0)	20.3 (1.0)	-19.4 (2.4)	12.5 (6.4)	11.1 (2.0)
hydrocodone	-27.2 (4.4)	-6.7 (3.5)	-7.4 (2.1)	-27.4 (2.0)	8.7 (3.7)	16.9 (1.3)	-9.0 (4.7)	13.7 (6.9)	8.2 (4.6)
methamphetamine	-15.7 (3.1)	-11.0 (1.7)	-8.9 (3.5)	-7.0 (3.1)	6.9 (1.1)	18.4 (0.4)	6.3 (3.5)	10.5 (6.4)	9.7 (3.4)
MDMA	-15.3 (1.6)	-7.7 (2.2)	-5.9 (1.5)	-7.1 (2.3)	11.3 (4.1)	17.7 (1.3)	8.3 (2.8)	15.4 (5.5)	13.1 (4.3)
ketamine	-14.2 (1.8)	-11.7 (2.4)	-11.8 (2.2)	-2.6 (2.1)	7.1 (3.7)	16.0 (2.1)	8.6 (2.7)	8.5 (6.0)	8.2 (2.1)
clenbuterol	-27.5 (2.0)	-12.8 (0.8)	-10.5 (1.1)	-29.4 (2.4)	9.1 (0.5)	13.3 (4.4)	-13.5 (5.1)	9.6 (6.7)	6.9 (1.8)
zolpidem	-13.5 (2.4)	-13.5 (1.2)	-9.8 (2.6)	-7.8 (2.7)	4.4 (2.6)	10.8 (1.8)	4.8 (5.0)	9.7 (4.7)	8.8 (2.8)
cocaine	-17.4 (2.6)	-17.6 (1.9)	-15.5 (3.7)	-9.1 (2.6)	6.4 (2.8)	12.5 (1.8)	2.1 (5.0)	9.2 (5.1)	6.8 (3.5)
LSD	-20.3 (2.2)	-17.3 (2.5)	-14.0 (3.4)	-13.1 (2.8)	1.3 (1.1)	8.6 (1.9)	-0.2 (4.1)	5.2 (4.8)	6.8 (1.6)
phencyclidine	-17.3 (3.3)	-17.5 (1.7)	-12.9 (1.5)	-16.7 (1.6)	-1.1 (3.2)	5.0 (1.0)	-5.3 (4.8)	3.9 (3.9)	1.2 (2.1)
propranolol	-25.0 (3.1)	-13.2 (3.1)	-12.9 (4.3)	-34.7 (3.4)	8.7 (6.2)	23.2 (3.5)	1.8 (3.6)	9.0 (5.3)	10.1 (3.0)
fentanyl	-7.0 (1.4)	-8.3 (1.5)	-10.1 (1.3)	-6.9 (2.8)	4.3 (3.4)	11.1 (1.6)	8.4 (3.4)	11.8 (4.9)	8.5 (1.2)
buprenorphine	-10.8 (5.9)	-6.3 (1.6)	-5.5 (3.0)	-8.8 (4.2)	11.6 (2.4)	20.7 (1.4)	9.1 (2.4)	13.0 (5.5)	11.0 (3.0)
alprazolam	-7.9 (4.3)	-4.2 (2.1)	-14.2 (5.0)	10.0 (4.3)	14.7 (5.1)	19.7 (6.1)	22.5 (6.0)	16.6 (5.5)	13.6 (7.0)
methadone	-20.3 (3.5)	-19.0 (0.7)	-17.1 (2.5)	-14.5 (4.0)	3.9 (2.3)	9.5 (1.7)	2.5 (2.9)	6.5 (5.3)	3.9 (3.9)
clonazepam	-9.2 (1.7)	-12.3 (3.1)	-12.6 (0.8)	-0.2 (3.6)	1.9 (6.5)	14.1 (5.4)	13.8 (3.2)	2.6 (3.4)	0.5 (2.0)
flunitrazepam	-6.5 (2.4)	-13.8 (1.8)	-7.2 (3.1)	4.3 (3.6)	0.7 (1.6)	12.7 (3.0)	11.1 (4.3)	1.5 (6.0)	8.4 (5.1)
canrenone	0.5 (2.7)	-4.4 (6.5)	-11.3 (2.0)	20.0 (5.0)	27.1 (1.5)	20.2 (7.8)	22.2 (9.2)	12.7 (1.5)	9.6 (5.8)
THC-COOH	-2.4 (1.1)	-3.5 (1.5)	-6.6 (0.9)	7.7 (2.7)	13.1 (2.4)	16.9 (1.9)	18.9 (6.2)	17.1 (3.8)	11.3 (2.2)
THC	-1.2 (0.5)	-5.3 (1.9)	-9.7 (2.0)	6.2 (3.8)	9.2 (3.9)	10.5 (1.8)	12.0 (4.0)	15.4 (6.5)	9.9 (4.5)

Corresponding relative standard deviation values [%] given in parentheses, substances arranged by retention order, n = 4.
